# Editorial: Emerging receptors as new targets in health and disease

**DOI:** 10.3389/fendo.2022.1073794

**Published:** 2022-11-16

**Authors:** Julie Dam, Julien Hanson, Martyna Szpakowska

**Affiliations:** ^1^ Institut Cochin, Inserm U1016, CNRS UMR 8104, Université Paris Cité, Paris, France; ^2^ Laboratory of Molecular Pharmacology, GIGA-Molecular Biology of Diseases, University of Liège, Liège, Belgium; ^3^ Laboratory of Medicinal Chemistry, Centre for Interdisciplinary Research on Medicines (CIRM), University of Liège, Liège, Belgium; ^4^ Immunopharmacology and Interactomics, Department of Infection and Immunity, Luxembourg Institute of Health, Esch-sur-Alzette, Luxembourg

**Keywords:** atypical receptors, elastin receptor complex, ACKR3, mineralocorticoid receptor, cardiovascular diseases, angiotensin receptor, bradykinin receptor

In this collection of Frontiers Research Topic, we have assembled an interesting series of reviews and articles on atypical receptors ranging from a nuclear receptor with a complex function (Rossier), an atypical GPCR activated by various ligands also functioning as a ligand scavenger (Duval et al.), a heteromeric GPCR complex that generates a new functional unit (Johnstone et al.), or an atypical heterotrimeric complex consisting of a transmembrane enzyme (Tembely et al.). These multiple regulatory systems illustrate the diversity of membrane receptors and their various modes of action. This complexity translates into the need to better understand the signalling systems of these receptors and to precisely delineate their functional role, which depends on cellular and tissue context, in order to identify their involvement in physiology and disease. The ultimate goal is to define or validate these receptors as efficient drug targets.

A complex dimension brought about by the G protein-coupled receptor (GPCR) family is that these receptors can assemble into homo- and heterodimers generating novel signalling platforms. Johnstone et al. describe an experimental approach based on energy transfer techniques to capture in HEK293 cells the dimerization of heterodimers between the angiotensin II type 2 receptor and the bradykinin type 2 receptor. In contrast to isolated receptors, heterodimerisation between these two receptors leads to novel G-protein coupling (here Gαz) suggesting new functionalities.

Vascular ageing results in the release of elastin-derived peptides (EDP or also known as elastokines) into the circulation, which are not inert but bioactive peptides that can bind to the elastin receptor complex (ERC). Tembely et al. discuss the nature of this atypical receptor complex, its signaling pathways and the pharmacological strategies that target it.

The mineralocorticoid receptor (MR) is a ligand-activated transcription factor belonging to the family of nuclear receptor binding steroid hormones. Its endogenous ligand, aldosterone, has numerous physiological effects, among which the reabsorption of sodium in the kidney is one of the most relevant in cardiovascular diseases. Thus, hyperaldosteronism leads to increased blood pressure and high risk of hypokaliemia. Several drugs have been developed to mitigate these effects. Beside their use in hypertension, the mineralocorticoid antagonists have been shown to have beneficial effects in heart failure. Hence, in addition to their anti-inflammatory role in the heart, these drugs have been proposed to reduce ventricular arrhythmias. In this issue, Rossier discusses the mechanistic link existing between MR and heart physiology. This insightful review nicely summarizes current knowledge and presents the evidences supporting the relevance of MR as a drug target in heart failure.

Another GPCR whose crucial role in pathophysiology of cardiovascular diseases has become evident over the years is the atypical chemokine receptor ACKR3 (formerly CXCR7). The unusual biology of this receptor lies with its inability to trigger conventional G protein signalling pathways as well as with its highly diverse ligand repertoire, including not only chemokines but also opioid peptides and proadrenomedullin-derived hormones critical for cardiovascular function. Duval et al. elegantly present the findings around ACKR3 in their historical context and discuss the implications of this receptor in atherosclerosis, myocardial infarction and stroke, highlighting the necessity to understand fully the molecular mechanisms controlling ACKR3-related functions to develop tailored therapeutics for cardiovascular diseases.

We hope that the reader will find in this Research Topic engaging illustrations of the diversity of membrane receptors and their potential as new targets for therapeutic strategies.

## Author contributions

All authors listed have made a substantial, direct, and intellectual contribution to the work and approved it for publication.

## Funding

This work was supported by the University of Liège, "Fonds pour la Recherche Scientifique" (F.R.S.-FNRS) Research Project (PDR T.0111.19) and Research Credit (CDR J.0083.2). JH is a F.R.S.-FNRS Senior Research Associate. JD was supported by the Agence Nationale de la Recherche (ANR-16-CE18-0013; ANR-21-CE14-0041), Fondation de France (#00107035 #00107081), Institut National de la Santé et de la Recherche Médicale (INSERM), Centre National de la Recherche Scientifique (CNRS) and the University Paris Cité. MS was supported by the Luxembourg Institute of Health (LIH), Luxembourg National Research Fund (INTER/FNRS grants 20/15084569) and F.R.S.-FNRS-Télévie (grants 7.4593.19, 7.4529.19, 7.8504.20 and 7.8508.22). JD, JH and MS are members of the COST Action ERNEST (CA18133), supported by COST (European Cooperation in Science and Technology, www.cost.eu.

## Conflict of interest

The authors declare that the research was conducted in the absence of any commercial or financial relationships that could be construed as a potential conflict of interest.

## Publisher’s note

All claims expressed in this article are solely those of the authors and do not necessarily represent those of their affiliated organizations, or those of the publisher, the editors and the reviewers. Any product that may be evaluated in this article, or claim that may be made by its manufacturer, is not guaranteed or endorsed by the publisher.

